# Candidate Genes Associated With Neurological Findings in a Patient With Trisomy 4p16.3 and Monosomy 5p15.2

**DOI:** 10.3389/fgene.2020.00561

**Published:** 2020-06-17

**Authors:** Thiago Corrêa, Fabiano Poswar, Bruno César Feltes, Mariluce Riegel

**Affiliations:** ^1^Post-Graduate Program in Genetics and Molecular Biology, Genetics Department, Universidade Federal do Rio Grande do Sul, Porto Alegre, Brazil; ^2^Medical Genetics Service, Hospital de Clínicas de Porto Alegre, Porto Alegre, Brazil; ^3^Department of Theoritical Informatics, Institute of Informatics, Universidade Federal do Rio Grande do Sul, Porto Alegre, Brazil

**Keywords:** Cri-du-chat, 4p16.3, *PPARGC1A*, *CTBP1*, *TRIO*, *TERT*, *CCT5*

## Abstract

In this report, we present a patient with brain alterations and dysmorphic features associated with chromosome duplication seen in 4p16.3 region and chromosomal deletion in a critical region responsible for Cri-du-chat syndrome (CdCS). Chromosomal microarray analysis (CMA) revealed a 41.1 Mb duplication encompassing the band region 4p16.3–p13, and a 14.7 Mb deletion located between the bands 5p15.33 and p15.1. The patient’s clinical findings overlap with previously reported cases of chromosome 4p duplication syndrome and CdCS. The patient’s symptoms are notably similar to those of CdCS patients as she presented with a weak, high-pitched voice and showed a similar pathogenicity observed in the brain MRI. These contiguous gene syndromes present with distinct clinical manifestations. However, the phenotypic and cytogenetic variability in affected individuals, such as the low frequency and the large genomic regions that can be altered, make it challenging to identify candidate genes that contribute to the pathogenesis of these syndromes. Therefore, systems biology and CMA techniques were used to investigate the extent of chromosome rearrangement on critical regions in our patient’s phenotype. We identified the candidate genes *PPARGC1A*, *CTBP1*, *TRIO*, *TERT*, and *CCT5* that are associated with the neuropsychomotor delay, microcephaly, and neurological alterations found in our patient. Through investigating pathways that associate with essential nodes in the protein interaction network, we discovered proteins involved in cellular differentiation and proliferation, as well as proteins involved in the formation and disposition of the cytoskeleton. The combination of our cytogenomic and bioinformatic analysis provided these possible explanations for the unique clinical phenotype, which has not yet been described in scientific literature.

## Background

Cri-du-chat syndrome (CdCS; OMIM #123450) is a genetic condition caused by a deletion in the short arm of chromosome 5. The phenotype is characterized by a cat-like cry, microcephaly, facial dysmorphism, psychomotor delays, and intellectual disability (Nguyen et al., 2015). Deletions, which occur at the end of the chromosome, as well as interstitial which result after two breaks, compose 80–90% of CdCS cases (Cerruti Mainardi, 2006). Unbalanced parental translocation occurs in approximately 10–15% of patients (Perfumo et al., 2000; Cerruti Mainardi, 2006). In addition, complex rearrangements, such as mosaicism, *de novo* translocation, or ring chromosomes, account for less than 10% of the cases (Perfumo et al., 2000). Wolf-Hirschhorn syndrome (WHS; OMIM #194190) is a contiguous gene deletion syndrome on the short arm of chromosome 4. It is characterized by facial dysmorphia, growth retardation, intellectual incapacity, and seizures (Zollino et al., 2008). However, duplication of the WHS critical region is a rare chromosomal condition causing mild clinical phenotypes, such as speech delay, facial dysmorphia, seizures, and delayed neuro and psychomotor development (Patel et al., 1995; Hannes et al., 2010; Carmany and Bawle, 2011; Cyr et al., 2011). However, the phenotypic and cytogenetic variability in affected individuals, such as the low frequency and the large genomic regions that can be altered, make it challenging to identify the candidate genes that contribute to the pathogenesis of these syndromes.

Here, we present an individual with duplication in the 4p16.3 region and deletion in the 5p15.2 region. The altered chromosomal segments are located in the critical regions of WHS and CdCS, respectively. This study reports a case never highlighted before in the literature. Systems biology and CMA were used to investigate the impact of chromosome rearrangement on critical regions in our patient’s phenotype.

## Case Presentation

**Figure 1:**
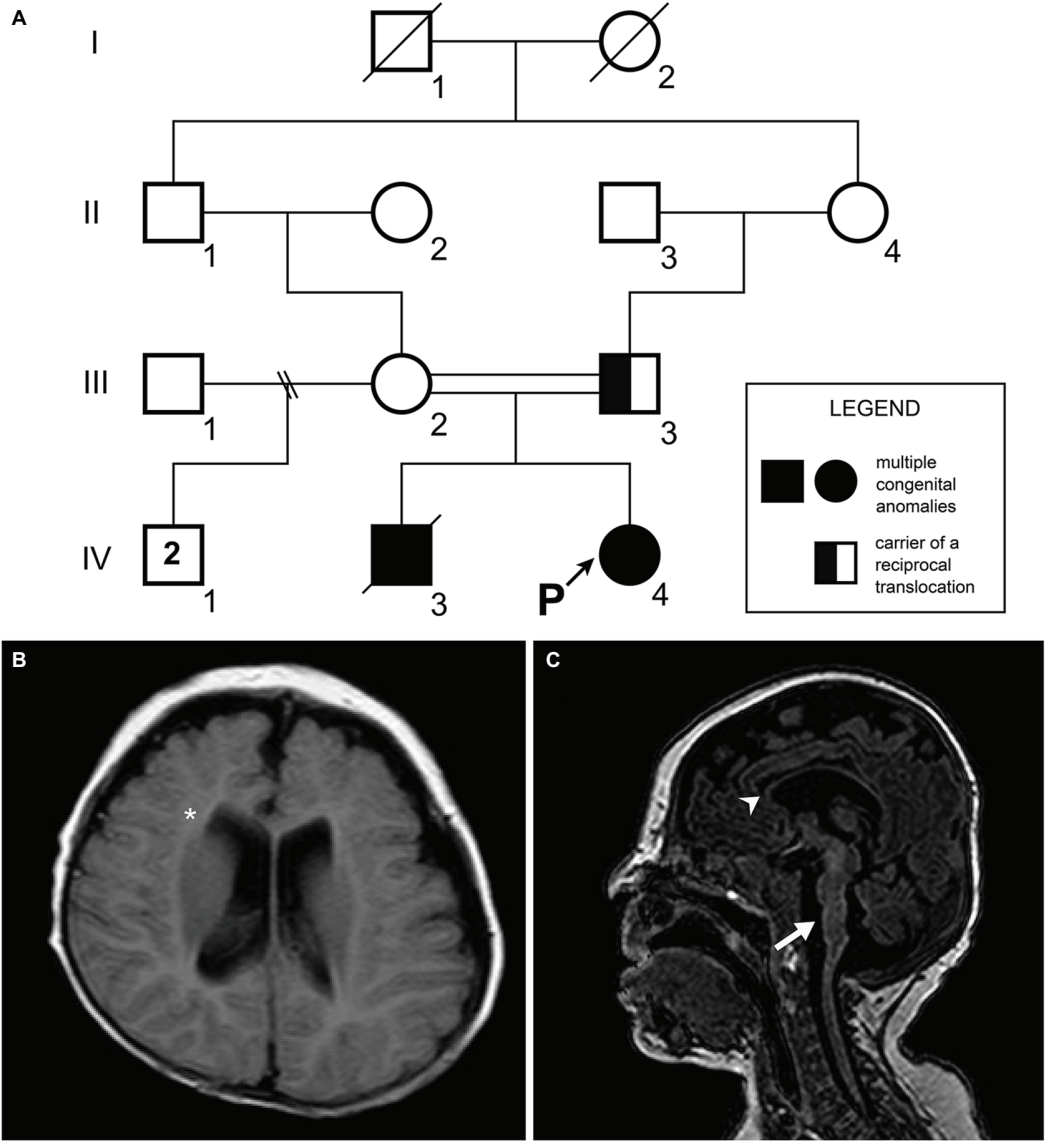
**(A)** Patient’s pedigree. A recessive trait was initially suspected on the basis of the parental consanguinity with recurrence in the offspring. The proband’s father (individual III.3) was a carrier of a balanced chromosomal translocation. **(B)** Transverse FLAIR image. Notice the white matter volume loss (asterisk). **(C)** Sagittal T1 weighted image showing thin corpus callosum (arrowhead) and pontine hypoplasia with dysgenesis of the cerebellar vermis (arrow).

A 5-day-old female was referred for investigation of congenital abnormalities such as imperforate anus and rectovaginal fistula, as well as atrial septal defect. Family history is noteworthy as it highlights consanguineous parents, and a brother who died with similar clinical presentation of imperforate anus, congenital heart defect, and clubfeet (Figure 1A). The pregnancy of the patient was uneventful, and the girl was born at home at the gestational age of 36 weeks, weighing 2,160 g, and a total length of 39 cm. On her first physical examination in our center, she had a low weight (2,045 g), down slanting palpebral fissures, short palpebral fissures, ptosis, widely spaced eyes, thin upper lip, clubfeet, overlapping fingers, micrognathia, and a high-pitched cry. Neurological examination was extraordinary as there was hypertonia of extremities and an absence of the Moro reflex. At the age of 1 month, the patient suffered seizure episodes with eye deviation that were controlled with phenobarbital drugs. In the electroencephalogram, acute wave discharges with multifocal distribution were observed in both hemispheres with predominance over the left temporal region. The brainstem illustrated that there was auditory potential; however, the scan showed abnormalities within the visual region. A brain MRI performed at the age of 5 months showed a thin corpus callosum, white matter volume loss, pontine hypoplasia, and dysgenesis of the cerebellar vermis (Figures 1B,C). Despite this, myelination was in accordance with her age. After being subjected to surgical procedures which had no complications, she was discharged at the age of 5 months and 25 days. Although the patient had a tracheostomy and a nasoenteral tube, she was, clinically, in a stable condition.

**Figure 2:**
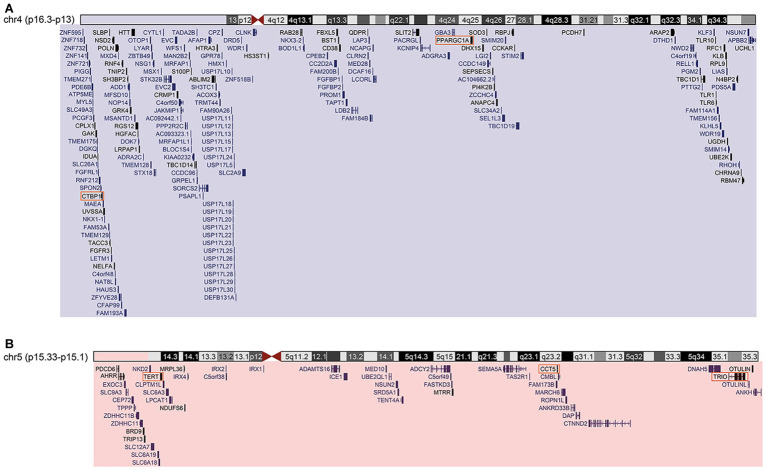
**(A)** Cytogenomic profile of the region of chromosome 4 duplication. Segment duplicated highlighted in blue rectangle. UCSC-Genome Browser-Dec.2013 (GRCh38/hg38) – genomic position/search term: chr4:71552-41263831 – track: GENCODE V29. **(B)** Cytogenomic profile of the deleted region of chromosome 5. Segment deleted highlighted in red rectangle. UCSC-Genome Browser-Dec.2013 (GRCh38/hg38) – genomic position/search term: chr5:269963-15032936 – track: GENCODE V29. Orange squares are genes considered H-B.

Karyotyping identified typical patterns of GTG bands in the mother (46,XX), and paternal reciprocal translocation with breakpoints in 4p16.3 and 5p15.2 regions [46,XY,t(4;5)(p16.3;p15.2)]. The proband was identified with 4p16.3–p13 trisomy and 5p15.33–p15.2 monosomy [46,XX,der(5) t(4,5)(p16.3;p15.2)pat]. Fluorescence *in-situ* hybridization (FISH) analysis confirmed three fluorescence signals for the 4p16.3 band, and only one fluorescence signal in the 5p15.2 proband. CMA revealed duplication in chromosome 4 (41.1 Mb) encompassing the bands 4p16.3–p13. The approximate genomic position was defined in chr4:71552–41263831 (GRCh38/hg38), comprising 198 genes (Figure 2A). Chromosome 5 was outlined with a deletion of 14.7 Mb located between the bands 5p15.33 and p15.1. The genomic position was estimated in chr5:269963–15032936 (GRCh38/hg38), comprising 50 genes (Figure 2B).

## Laboratory Investigations

### Cytogenetic Studies

Karyotyping was performed on metaphase spreads prepared from peripheral blood samples. The chromosomal analysis was conducted through GTG banding at a 550-band resolution, and at least 100 cells were analyzed. FISH experiments were performed following standard techniques with commercially available locus-specific probes such as a dual-color commercial probe for the CdCS and WHSCR (Cytocell, UK). The *CTNND2* probe for 5p15.2 (red spectrum) contains a sequence homologous to the D5S2883 locus and covers approximately 159 kb of this locus. The probe for the 4p16.3 (red spectrum) contained a sequence that was homologous to the D4S166 locus and covered approximately 223 kb of this locus. At least 30 cells were analyzed per hybridization. The sample was mapped using CMA, using a 60-mer oligonucleotide-based microarray with a theoretical resolution of 40 kb (8 × 60 K, Agilent Technologies Inc., Santa Clara, CA, USA). The arrays were analyzed using a microarray scanner (G2600D) and feature extraction software (version 9.5.1, Agilent Technologies). The images were analyzed using Cytogenomics v2.0 and v2.7 with the statistical algorithm ADM-2 and a sensitivity threshold of 6.0.

### Network Design

The protein-protein interaction (PPI) metasearch engine STRING 11.0 (http://string-db.org/) was used to create PPI networks based on deleted or duplicated genes located in the altered chromosomal regions. CMA, with a subsequent search in the UCSC genome browser of the human genome assembly (December 2013), retrieved 591 genes and predicted genes belonging to the duplicate area, as well as 246 from the deleted region (Kent et al., 1976; von Mering et al., 2005). The parameters used in STRING were: (i) degree of confidence, 0.400; (ii) 500 proteins in the first and second shell; and (iii) methods used were neighborhood, experiments, databases, and co-occurrence. The final PPI network was obtained through STRING and analyzed using Cytoscape 3.7.0 (Shannon et al., 2003).

### GO and Centralities Analysis

The Gene Ontology (GO), Kyoto Encyclopedia of Genes and Genomes (KEGG), and Reactome libraries were searched using the ClueGO Cytoscape plugin (Bindea et al., 2009). Significant GO predictions were selected based on a *p* ≤ 0.05, with the Bonferroni family-wise false discovery rate (FDR) test. Node degree and betweenness centralities were measured to identify hub-bottleneck (H-B) nodes from the PPI network using the Cytoscape plugin and CentiScaPe 3.2.1 (Scardoni et al., 2009).

### Molecular Pathway Reconstruction

The PathLinker Cytoscape plugin was used to identify and reconstruct possible signaling pathways of interest from our PPI network (Murali et al., 2017). PathLinker computes the *k* shortest paths that connect any source to any target in the network, and subsequently generates a subnetwork. It also creates a table with a rank of the shortest paths (Murali et al., 2018). The deleted gene network in the Cri-du-chat region (CdCR-Net) was used as a background, and the H-B CCT5, TERT, and TRIO were used as a source and targets for paths calculations. The parameters used in PathLinker were: (i) k: 50 (number of paths the user seeks); (ii) edge penalty: 1; and (iii) edge weight: weight probabilities, whereby it considers the edge weights as multiplicative, which result in the *k* highest cost paths (Murali et al., 2017).

## Discussion

Here, we have presented a patient with brain alterations and dysmorphic features resulting from chromosomal deletion in the critical region related to CdCS and duplication in the critical region related to WHS. The patient’s clinical findings overlap with previously reported cases of both 4p duplication syndrome and CdCS (Table 1). Overall, the patients presentation is notably similar to CdCS patients as she presented with a weak, high-pitched voice and also showed similar pathogenicity observed in the brain MRI. Furthermore, the patient’s anorectal malformations are also similar to what can be observed in certain cases of CdCS (Marcelis et al., 2011). Nevertheless, she presents with some features that are common to both conditions discussed, or those more frequently described in patients with abnormalities of the critical region of WHS.

**Table 1:** Comparison of the clinical manifestations of this patient, and previously reported patients with Cri-du-chat syndrome and Trisomy 4p syndrome.

Clinical manifestations	This patient	Cri-du-chat patients (Honjo, 2018; Mainardi, 2001)^*^	Trisomy 4p patients (Patel et al., 1995; Dallapiccola, 1977)^*^^,^^**^
Imperforate anus	Present	−	−
Preterm birth	Present	+	+
Micrognathia	Present	++	+
Low birth weight	Present	+	+
Psychomotor retardation	Present	++	++
Downslanting palpebral fissures	Present	++	++
Widely spaced eyes	Present	++	+
Abnormalities of the fingers	Present	++	++
Prominent heels	Present	−	++
Weak, high-pitched voice	Present	++	−
Growth deficiency	Present	++	++
Seizures	Present	+	+
Microcephaly	Present	++	++
Pontine hypoplasia	Present	++	−

++, presence of the manifestation in 50% or more of the patients; +, presence of the manifestation in more than 10%, but less than 50% of the patients; −, not frequently reported.

* Based on overall reported frequencies in patients with variable chromosomal breakpoints.

** Most previously reported trisomy 4p patients also have other chromosomal imbalances and variable breakpoints.

To identify possible candidates that could help explain this scenario, a centrality analysis was carried out to identify H-B. These proteins represent nodes with high degree and betweenness scores, which are frequently related to the control of information flow between groups of proteins with central functions in a biological network (Hahn and Kern, 2005; Scardoni et al., 2009).

Two H-B were identified in the WHR-Net (Supplementary Figure S1A). The H-B PPARGC1A is a transcriptional coactivator of a subset of genes related to oxidative phosphorylation, which regulate glucose and lipid metabolism, mitochondrial biogenesis, and muscle fiber development (Terada et al., 2002; Tunstall et al., 2002; Puigserver and Spiegelman, 2003; Finck et al., 2006). As expected, and through the enrichment analysis, PPARGC1A was found to be associated with the regulation of progesterone synthesized in the biosynthetic pathway (Supplementary Figure S1B). The deregulation of transcription and mitochondrial function caused by PPARGC1A is associated with conditions such as amyotrophic lateral sclerosis, Parkinson’s disease, Alzheimer’s disease, and Huntington’s disease (Weydt et al., 2006; Eschbach et al., 2013; Jesse et al., 2017). Additionally, the second H-B, CTBP1 plays a role in the regulation of gene expression during embryonic development, as well as participation in axial patterning and cellular proliferation and differentiation (Hildebrand and Soriano, 2002; Van Hateren et al., 2006). A *de novo* heterozygous missense mutation in the *CTBP1* (R331W) causes hypotonia, developmental delay, ataxia, and intellectual disability (Beck et al., 2016, 2019). As heterozygous null variants of *CTBP1* are commonly found in unaffected individuals, gain of function rather than loss of function mechanisms are more likely to be associated with these clinical findings (Beck et al., 2019). Moreover, *PPARGC1A* and *CTBP1* are duplicated in the 4p region in the patients with neuropsychomotor delay, intellectual disability, and speech delay (Figure 2A; Cotter et al., 2001; Paskulin et al., 2009; Carmany and Bawle, 2011). Consequently, topological analysis indicates that the increased dosage of the *PPARGC1A* and *CTBP1* genes may have contributed to the neuropsychomotor delay and neurological alterations found in our patient (Table 1).

TRIO, TERT, and CCT5 were identified as H-B in the CdCR-Net (Supplementary Figure S2A). TRIO has functions in cell migration and morphogenesis during cerebellum development, including neurite and axon outgrowth (Briancon-Marjollet et al., 2008; Peng et al., 2010; Tao et al., 2019). *Trio* knockout causes reduction in the extension of granule neurons from the cerebellum and severe ataxia in mice (Peng et al., 2010). Furthermore, the *TRIO* haploinsufficiency in mice increases anxiety; impairs sociability and motor coordination, disrupts learning capacity and spatial memory, and decreases brain and neuron size (Zong et al., 2015; Katrancha et al., 2019). In this sense, the hemizygosity of *TRIO* may have contributed to the clinical findings in our patient at the age of 5 months, such as the thin corpus callosum, white matter volume loss, pontine hypoplasia, and dysgenesis of the cerebellar vermis (Figures 1B,C).

Moreover, damages in spatial memory are associated with TERT as its knockout in the hippocampus of adult mice impairs spatial memory processes during neural development (Zhou et al., 2017). The deficiency of *TERT* may also result in microvascular dysfunction in mice (Ait-Aissa et al., 2018). Furthermore, we found that TERT was associated with the negative regulation of apoptotic processes of endothelial cells in GO analysis (Supplementary Figure S2B). In addition, TERT shows interaction with CCT5 in the Y2H library screen (Wang et al., 2013). The H-B CCT5 is involved in cilia morphogenesis and survival of sensory neurons (Posokhova et al., 2011). Mutations in this gene may cause neurodegenerative diseases, such as spastic paraplegia and sensory neuropathy (Bouhouche et al., 2006; Pavel et al., 2016; Pereira et al., 2017). Additionally, *TERT* and *CCT5*, located in the critical region of CdCS, are associated with microcephaly and intellectual disability, reported in patients from several other studies (Figure 2B; Cerruti Mainardi, 2006). In this sense, deletion of *TERT* and *CCT5* genes could be involved with psychomotor retardation and microcephaly as presented in the present case (Table 1).

**Figure 3:**
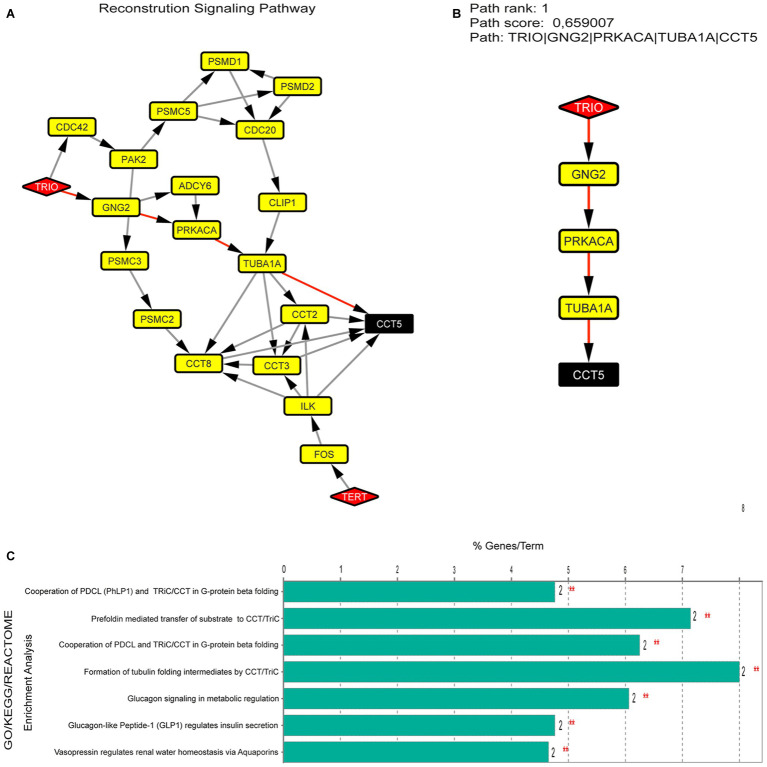
**(A)** Signaling pathway identified between H-B (TRIO, CCT5, and TERT), using the Pathlinker plugin. **(B)** Pathway better ranked by path score between 50 possible pathways. **(C)** Enrichment analysis in proteins present in the pathway (TRIO, GNG2, PRKACA, TUBA1A, and CCT5).

To investigate the importance of the H-B from CdCR-Net and their associated pathways (Figure 3A), we identified TRIO, GNG2, PRKACA, TUBA1A, and CCT5 as having the highest path score (Figure 3B). These proteins are involved in signaling mechanisms, including differentiation and proliferation, as well as roles in the formation and disposition of the cytoskeleton (Yajima et al., 2012; Tseng et al., 2017). In the latter case, TRIO, TUBA1A, and CCT5 play roles in the folding of actin and tubulin; reorganization; and assembly of the cytoskeleton during migration, growth, and differentiation of neurons (Seipel et al., 1999; Tian et al., 2010; Tracy et al., 2014). Genes that contribute to a common disorder tend to share core bioprocesses (Figure 3C; Goh et al., 2007). For instance, the chaperonin complex, CCT, which is also formed by the subunit CCT5, facilitates the formation of the heterodimeric form of the G-protein gamma subunits, similar to the GNG2 protein (Lukov et al., 2005). The formation of tubulin folding intermediates is also produced by CCT, in which unfolded actins and tubulins, such as TUBA1A are transferred to cytosolic chaperonin CCT (Frydman et al., 1992; McCormack et al., 2001). Interestingly, mutations or loss function of *TRIO*, *TUBA1A*, and *CCT5* is associated with intellectual disability, defects in dendritic branching, synapse function, sensory neuropathy, and microcephaly in humans (Bouhouche et al., 2006; Morris-Rosendahl et al., 2008; Kumar et al., 2010; Ba et al., 2016; Pavel et al., 2016; Pengelly et al., 2016; Belvindrah et al., 2017).

Essential human genes are expected to encode central proteins, such as the H-B genes, and be expressed in different tissues (Goh et al., 2007; Loscalzo and Barabasi, 2011). The haploinsufficiency of the H-B genes observed in our PPI-network could affect pathways related to the cilia morphogenesis, dendritic branching, and synapse function, including neurite and axon outgrowth, which consequently could have led to the neurodevelopment delay and microcephaly observed in our patient. In addition, the identification of CTBP1, PPARGC1A, CCT5, TERT, and TRIO with different approaches brought new insights on the pathogenesis involved in these rare chromosomal rearrangements, such as those presented here, in a case never reported before.

## Data Availability Statement

All datasets generated for this study are included in the article/Supplementary Material.

## Ethics Statement

The study includes a statement on ethics approval and consent. The study was approved by the Ethics in Research Committee of Hospital de Clínicas de Porto Alegre (HCPA), under the reference number 10-560. Written informed consent form was obtained from the guardians of the participant for the publication of this paper.

## Author Contributions

TC, BF and MR conceived, designed the study and analyzed all the data. FP analyzed the clinical data. All authors contributed to the writing manuscript. MR revised the manuscript.

## Conflict of Interest

The authors declare that the research was conducted in the absence of any commercial or financial relationships that could be construed as a potential conflict of interest.

## Supplementary Material

The Supplementary Material for this article can be found online at: https://www.frontiersin.org/articles/10.3389/fgene.2020.00561/full#supplementary-material.
