# Supplementary material for: Candidate Genes Associated With Neurological Findings in a Patient With Trisomy 4p16.3 and Monosomy 5p15.2
Source: Front Genet. 2020 Jun 17;11:561. doi: 10.3389/fgene.2020.00561 (PMC7311770; doi:10.3389/fgene.2020.00561)
Supplement: Supplementary file 1 [file Data_Sheet_1.PDF]

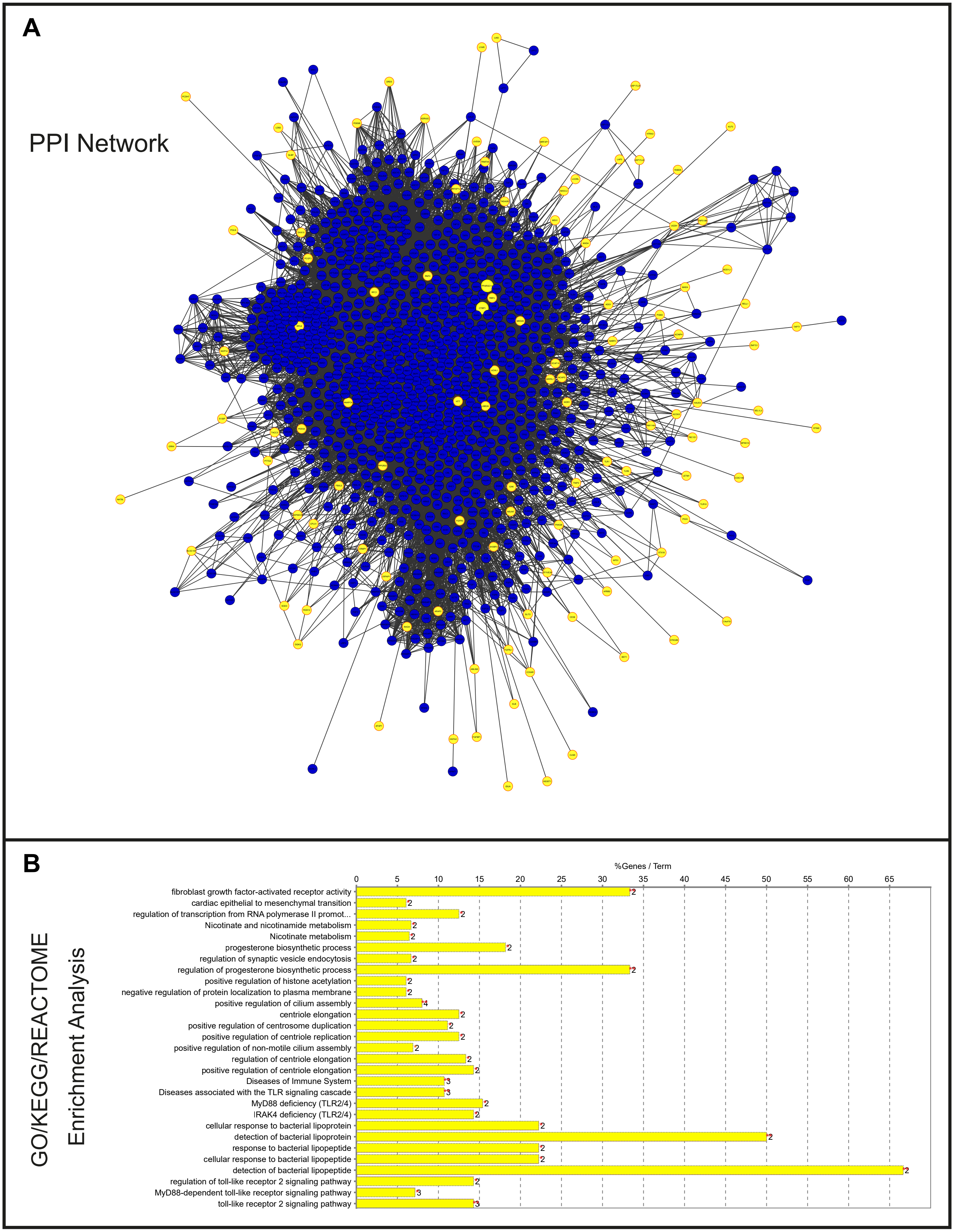


**Fig.S1.** (A) The PPI network. List of 591 genes and gene predictions were obtained from GENCODE V29 GRCh38/hg38-UCSC database. Interaction data from STRING were used to construct networks using Cytoscape software. The network is composed of 1113 nodes and 26085 edges. Black nodes with orange border are target proteins encoded by duplicated genes (seed genes). Black nodes with yellow border are H-B (PPARGC1A and CTBP1). (B) Functional enrichment analysis to duplicated genes using ClueGO plugin.
