# Supplementary material for: Candidate Genes Associated With Neurological Findings in a Patient With Trisomy 4p16.3 and Monosomy 5p15.2
Source: Front Genet. 2020 Jun 17;11:561. doi: 10.3389/fgene.2020.00561 (PMC7311770; doi:10.3389/fgene.2020.00561)
Supplement: Supplementary file 2 [file Data_Sheet_2.PDF]

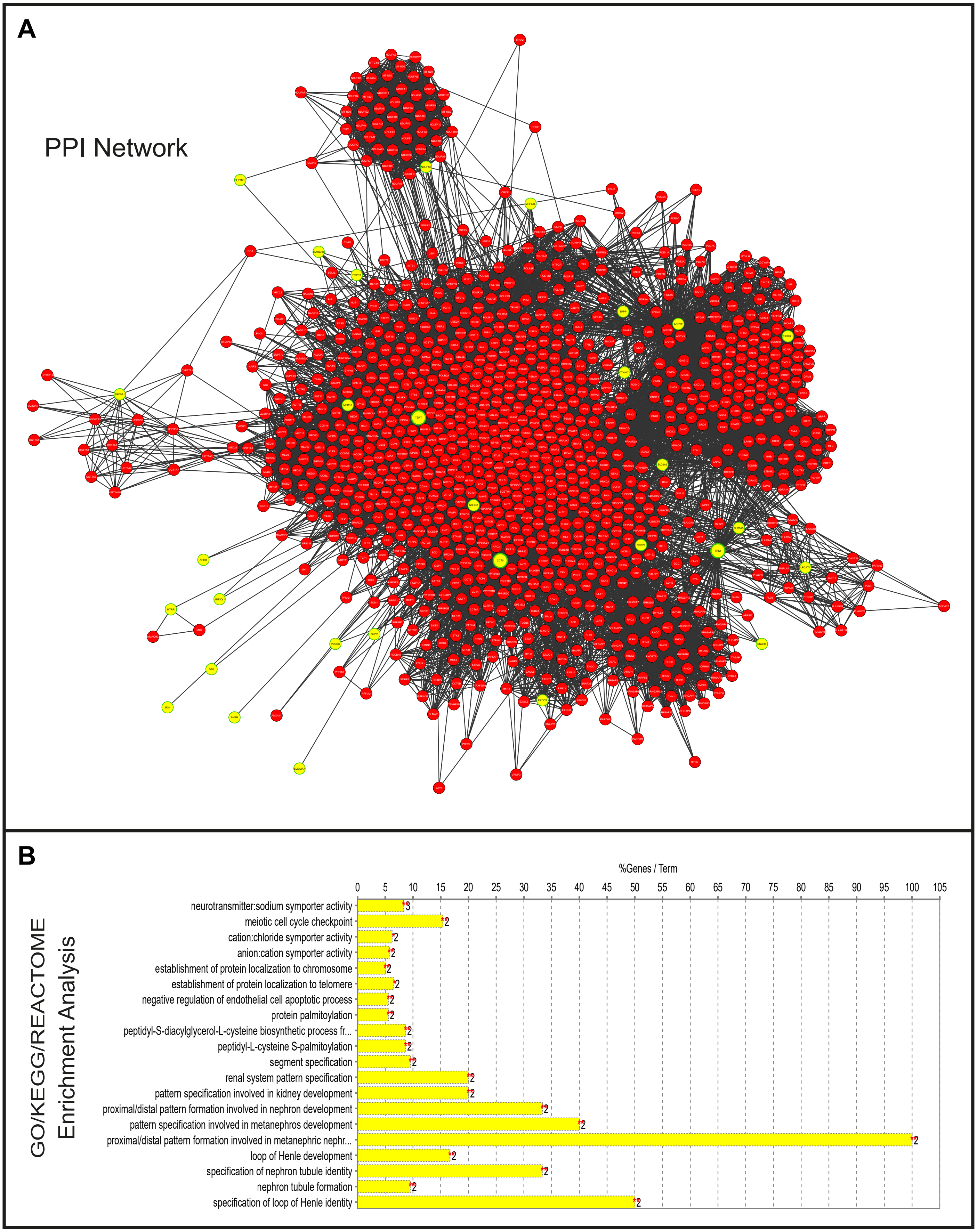

Fig.S2. (A) The PPI network. List of 246 genes and gene predictions were obtained from GENCODE V29 GRCh38/hg38-UCSC database. Interaction data from STRING were used to construct networks using Cytoscape software. The network is composed of 1030 nodes and 24748 edges. Black nodes with green border are target proteins encoded by deleted genes (seed genes). Black nodes with yellow border are H-B (CCT5, TERT and TRIO). (B) Functional enrichment analysis to deleted genes using ClueGO plugin. Only bioprocess with p-corrected value ≤ 0.05 were considered.
